# Serum *β*2-microglobulin may be a viral biomarker by analyzing children with upper respiratory tract infections and exanthem subitum: a retrospective study

**DOI:** 10.7717/peerj.11109

**Published:** 2021-04-06

**Authors:** Xulong Cai, Qiaolan Xu, Chenrong Zhou, Tongjin Yin, Li Zhou

**Affiliations:** 1 Department of Pediatrics, Yancheng Third People’s Hospital, Yancheng, China; 2 Department of Pediatrics, The Yancheng School of Clinical Medicine of Nanjing Medical University, Yancheng, China

**Keywords:** Serum β2-microglobulin, Upper respiratory tract infections, Exanthem subitum, Viral biomarker

## Abstract

**Background:**

Due to the lack of effective and feasible viral biomarkers to distinguish viral infection from bacterial infection, children often receive unnecessary antibiotic treatment. To identify serum *β*2-microglobulin that distinguishes bacterial upper respiratory tract infection from viral upper respiratory tract infection and exanthem subitum in children.

**Methods:**

This retrospective study was conducted from January 1, 2019 to September 30, 2020 in Yancheng Third People’s Hospital. Children with upper respiratory tract infection and exanthem subitum were recruited. The concentration of serum *β*2-microglobulin in the viral and bacterial infection groups were statistically analyzed.

**Results:**

A total of 291 children included 36 with bacterial upper respiratory tract infection (median age, 13 months; 44.4% female), 197 with viral upper respiratory tract infection (median age, 12 months; 43.7% female) and 58 with exanthem subitum (median age, 13 months; 37.9% female). When the concentration of *β*2-microglobulin was 2.4mg/L, the sensitivity to distinguish viral from bacterial upper respiratory tract infection was 81.2% (95% CI [75.1–86.4%]), and the specificity was 80.6% (95% CI [64.0–91.8]%). When the cutoff was 2.91 mg/L, the sensitivity of *β*2-microglobulin to distinguish exanthem subitum from bacterial upper respiratory tract infection was 94.8% (95% CI [85.6–98.9]%), and the specificity was 100% (95% CI [90.3–100]%).

**Conclusions:**

Serum *β*2-microglobulin may be a significant biological indicator in children with upper respiratory tract infection and exanthem subitum.

## Introduction

Upper respiratory tract infection (URTI) is a common disease in children. Children with URTI often go to the outpatient or emergency department because of fever. In addition to fever, the clinical manifestations of children with URTI include runny nose, sneezing, hoarseness, pharyngeal congestion, mild dry cough and swelling of tonsil (Weintraub, 2015). The early manifestation of exanthem subitum is similar to acute URTI. The early main symptoms of exanthem subitum is persistent high fever, and the clinical symptoms of runny nose, pharyngeal congestion and tonsil swelling will also appear (Robert Kliegman, 2019; Stone, Micali & Schwartz, 2014). Exanthem subitum usually depends on the characteristics of rash appearance after the disappearance of fever for retrospective diagnosis (Stone, Micali & Schwartz, 2014). The age of children with exanthem subitum is mostly between 6 months and 24 months (Hattori et al., 2019). It is challenging to distinguish acute URTI from early stage exanthem subitum according to clinical manifestations and symptoms.

Most of the pathogens of acute URTI are viruses (Wei et al., 2017). There are also a small number of bacteria (Bellussi et al., 2019). Common virus infection (adenovirus, Parainfluenza virus and respiratory syncytial virus) only need symptomatic treatment (Tang et al., 2019). Influenza virus infection should be treated with anti influenza drugs. Antibiotics are required for bacterial infections. However, there is an obvious overuse of antibiotics in children with viral URTI (Cheng et al., 2019).

Exanthem subitum caused by human herpesviruses 6 and 7 usually recovers well and only needs symptomatic treatment (Stone, Micali & Schwartz, 2014). Exanthem subitum is often accompanied by neutropenia and leucopenia (Arnež et al., 2016). Sepsis can also be characterized by fever and neutropenia (Das, Trehan & Bansal, 2018; Dien Bard & Mongkolrattanothai, 2019). Therefore, persistent high fever, neutropenia and leucopenia in the early stage of exanthem subitum can easily lead to misdiagnosis, such as URTI and sepsis. And then, there may be antibiotic abuse.

A study found that *β*2-microglobulin (*β*2-MG) expression was closely related to cytotoxic T cells in a mouse model (Zijlstra et al., 1990). Interestingly, the activation of cytotoxic T cells participate in the immune response to viral infection (Baugh, Tzannou & Leen, 2018; David et al., 2019; Langellotti et al., 2020). A previous study found that *β*2-MG was significantly increased in viral lower respiratory tract infection, and it may distinguish viral infection from bacterial infection (Cai et al., 2020). This study analyzed the distribution characteristics of *β*2-MG in children with exanthem subitum and acute URTI. We explored the theoretical basis of identification of viral infection by *β*2-MG.

## Methods

### Study population

The subjects were children who visited to our hospital between January 1, 2019 and September 30, 2020. We collect information by browsing the electronic medical record. This study conducted a retrospective study on acute URTI and exanthem subitum.

Inclusion criteria: (1) Acute URTI and exanthem subitum were diagnosed by experienced specialists. (2) The age of children with exanthem subitum is mainly between 6 months and 24 months. We collected children between 6 months and 24 months. (3) Each case was infected with only one pathogen. (4) Serum *β*2-MG detection had been completed in the acute stage of acute URTI and in the febrile period of exanthem subitum.

Exclusion criteria: (1) Combined with other infectious diseases. (2) Children have immune deficiency and hereditary diseases. (3) There was kidney disease. Because *β*2-MG is affected by renal filtration rate. (4) The children had used immunosuppressive drugs in the last two weeks.

The project was approved by the ethics committee of the Yancheng Third People’s Hospital (Approval Number: 2019100). Informed consent was waived by the ethics committee of the Yancheng Third People’s Hospital because the study was considered to pose the least risk to participants.

### Identification of pathogen

All cases were examined for viral and bacterial pathogens. Common viral pathogens were detected, including influenza A virus, influenza B virus, adenovirus, respiratory syncytial virus, parainfluenza virus and human herpesviruses 6. Viral infection: (1) The identification of respiratory virus is through antigen detection or PCR nucleic acid detection of nasopharynx swab samples. (2) The infection of human herpesviruses 6 was confirmed by PCR nucleic acid detection. Bacterial infection: it was identified by pharyngeal secretion culture or blood culture.

### Statistical analysis

The mean ± standard deviation or interquartile ranges was used to represent the characteristics of continuous variables. Frequency and percentage were used to describe the features of categorical variables. Student’s *t*-test was used to analyze the characteristics of continuous variables in two groups. Receiver operating characteristic (ROC) curves was used to assess sensitivity and specificity. *P* value less than 0.05 indicates statistical significance. All statistical analyses were performed by SPSS 24 software or GraphPad Prism V5.

## Results

### General characteristics of subjects

**Figure 1:**
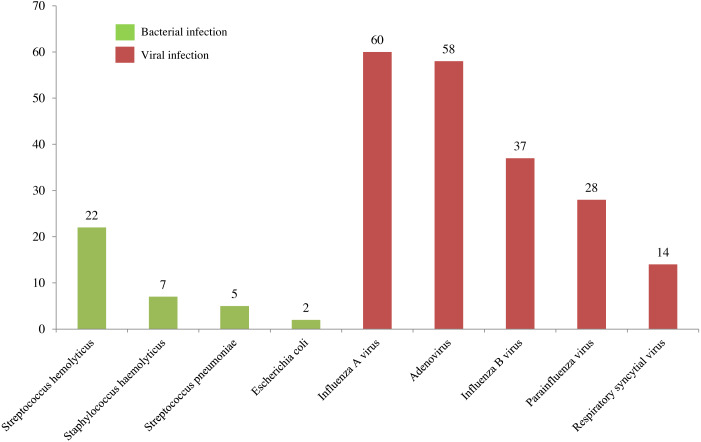
Pathogens of upper respiratory tract infection. In the cases of upper respiratory tract infection, there are 36 cases of bacterial infection and 197 cases of viral infection. The children’s ages range from 6 months to 24 months. The main pathogen of bacterial URTI is Streptococcus hemolyticus. The main pathogens of viral URTI are influenza A virus and adenovirus.

A total of 291 children were collected for analysis. There were 36 cases of bacterial URTI, 197 cases of viral URTI and 58 cases of exanthem subitum. Fever is the common nonspecific symptom of acute URTI and exanthem subitum. Exanthem subitum have symptoms similar to acute URTI, such as runny nose (13.8%), pharyngeal congestion (29.3%), tonsil swelling (10.3%), mild dry cough (12.1%) (Table 1). The main pathogen of bacterial URTI is Streptococcus hemolyticus (Fig. 1). The main pathogens of viral URTI are influenza A virus and adenovirus.

**Table 1:** General characteristics of subjects.

Characteristic	Upper respiratory tract infection		Exanthem subitum *n* = 58
	Bacterial *n* = 36	Viral *n* = 197	
Female, no. (%)	16(44.4)	86(43.7)	22(37.9)
Age Median (IQR), months	13(11,19)	12(11,16)	13(10,15)
Fever, no. (%)	34(94.4)	188(95.4)	58(100)
Peak temperature (Mean ± SD), °C	39.0 ± 0.6	39.2 ± 0.5	39.5 ± 0.5
Duration of fever (Mean ± SD), day	3.6 ± 2.5	3.4 ± 1.5	4.5 ± 1.1
Febrile convulsion, no. (%)	4(11.1)	31(15.7)	7(12.1)
Eating less, no. (%)	9(25.0)	44(22.3)	23(39.7)
Runny nose, no. (%)	21(58.3)	178(90.4)	8(13.8)
Pharyngeal congestion, no. (%)	36(100)	197(100)	17(29.3)
Swelling of tonsil, no. (%)	22(61.1)	72(36.5)	6(10.3)
Mild dry cough, no. (%)	2(5.6)	28(14.2)	7(12.1)

**Notes.**

IQR, quartile range.

### Characteristics of *β*2-MG in viral and bacterial URTI

**Figure 2:**
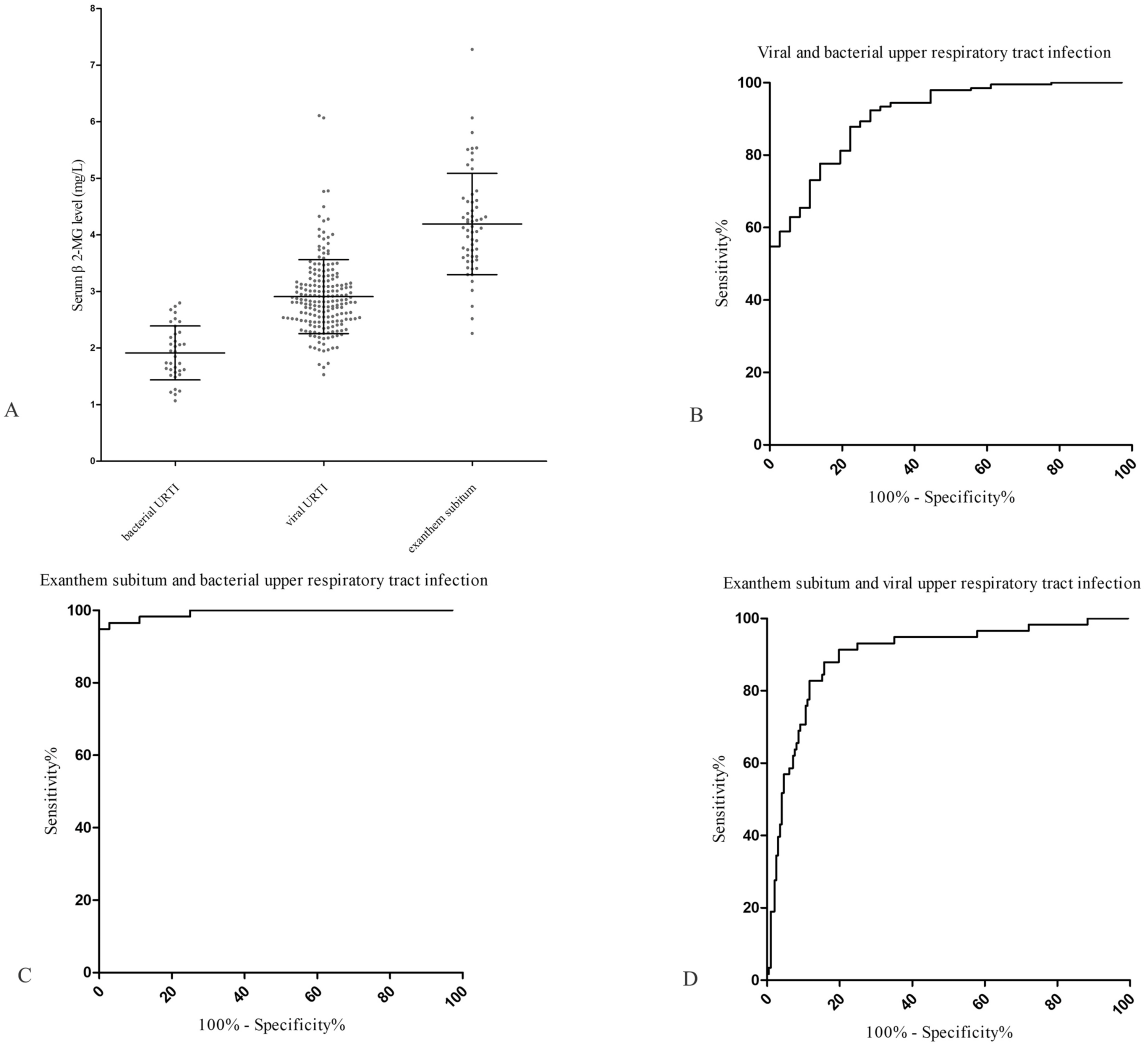
*β*2-MG for identification of viral infection by ROC curve evaluation. (A) The distribution of serum levels of *β*2-microglobulin in bacterial URTI, viral URTI and exanthem subitem groups are shown by scatter plot. (B) ROC curves of *β*2-microglobulin levels for differentiating viral from bacterial URTI. The AUC for *β*2-microglobulin was 0.91 (95% CI [0.86–0.96]). (C) ROC curves of *β*2-microglobulin levels for distinguishing exanthem subitem from bacterial URTI. The AUC for *β*2-microglobulin was 0.99 (95% CI [0.98–1.00]). (D) ROC curves of *β*2-microglobulin levels for distinguishing exanthem subitem from viral URTI. The AUC for *β*2-microglobulin was 0.90 (95% CI [0.85–0.95]).

Compared with bacterial infection group, serum *β*2-MG level was higher in viral URTI and exanthem subitum (Fig. 2A and Table 2). Sensitivity and specificity were calculated and analyzed by GraphPad Prism V5 on November 4, 2020. The area under the curve (AUC) for *β*2-MG was 0.91 (95% CI [0.86–0.96]) for discriminating viral from bacterial URTI (Fig. 2B). When the concentration of *β*2-MG is 2.4 mg/L, the sensitivity to distinguish viral from bacterial infection is 81.2% (95% CI [75.1–86.4]%), and the specificity is 80.6% (95% CI [64.0–91.8]%). Adenovirus infection was similar to bacterial infection, most of which were characterized by increased white blood cell count and C-reactive protein (Table S1). For *β*2-MG, the AUC was 0.88 (95% CI [0.82–0.95]) for discriminating adenovirus infection from bacterial infection (Fig. S1). *β*2-MG at cutoff 2.20 mg/L, the sensitivity to distinguish adenovirus infection from bacterial infection is 89.66% (95% CI [78.8–96.1]%), specificity is 72.2% (95% CI [54.8–85.8]%).

**Table 2:** Difference of *β*2-MG in viral and bacterial infections.

Variable	N	*β*2-microglobulin (mg/L)	*P* value
Bacterial URTI	36	1.92 ± 0.48	reference
Viral URTI	197	2.91 ± 0.65	<0.001
Exanthem subitum	58	4.19 ± 0.90	<0.001

**Notes.**

URTI, upper respiratory tract infection

The AUC for *β*2-MG was 0.99 (95% CI [0.98–1.00]) for discriminating exanthem subitum from bacterial URTI (Fig. 2C). When the cutoff was 2.91 mg/L, the sensitivity of *β*2-MG to distinguish exanthem subitum from bacterial URTI was 94.8% (95% CI [85.6–98.9]%), and the specificity was 100% (95% CI [ 90.3–100]%).

### Difference of *β*2-MG in exanthem subitum and virus URTI

The concentration of serum *β*2-MG in viral URTI and exanthem subitum was increased. Further statistical analysis, the level of *β*2-MG in exanthem subitum was significantly higher than that of viral URTI (4.19 ± 0.90 mg/L vs 2.91 ±  0.65 mg/L, *P* < 0.001). The AUC for *β*2-MG was 0.90 (95% CI [0.85–0.95]) for discriminating exanthem subitum from viral URTI (Fig. 2D). When the cutoff was 3.40 mg/L, the sensitivity of *β*2-MG to distinguish exanthem subitum from viral URTI was 87.93% (95% CI [76.7–95.0]%), and the specificity was 84.3% (95% CI [78.4–89.1]%).

## Discussion

Through the analysis of the general characteristics of acute URTI and exanthem subitum, our results suggested that the clinical manifestations of some children with exanthem subitum were similar to acute URTI. Exanthem subitum may be difficult to distinguish from acute URTI before the rash appears. Although most of acute URTI are viral infections, there are also some bacterial infections (Hersh et al., 2013). Bacterial URTI requires antibiotic treatment. However, the overuse of antibiotics in children with acute upper respiratory tract infection is serious (Cheng et al., 2019; Kuchar et al., 2015; Trinh et al., 2020).

HHV-6 infection is the main cause of exanthem subitum (Agut, Bonnafous & Gautheret-Dejean, 2016). In addition, there is a small part of exanthem subitum is caused by HHV-7. Exanthem subitum only needs symptomatic treatment. Although the prognosis of exanthem subitum is benign, it is challenging to make a diagnosis before eruption. When the early persistent high fever can not be diagnosed in children with exanthem subitum, parents are anxious and reasonable treatment is facing challenges. Children with persistent high fever and without an apparent source are more likely to use unnecessary antibiotics before the viruses are identified (Colvin et al., 2012).

Identification of pathogens is helpful for early diagnosis of diseases. At present, there are some ways to identify pathogens. Culture is the most specific method to confirm pathogens infection, but it is low sensitivity, expensive and time-consuming (Agut, Bonnafous & Gautheret-Dejean, 2015). It has a long time span to diagnose pathogens by the change of serum antibody titer in acute phase and convalescent stage, which is suitable for retrospective diagnosis. PCR nucleic acid detection has high sensitivity and specificity, which is helpful to identify pathogens (Korman, Alikhan & Kaffenberger, 2017). However, the cost is expensive, so the PCR detection method has its limitations (Agut, Bonnafous & Gautheret-Dejean, 2015).

Acute URTI and exanthem subitum are common diseases in children. It is easy to identify pathogens in hospitals with advanced equipment. However, in the primary care hospitals, there is no perfect equipment for pathogen identification, especially PCR nucleic acid detection. In addition to pathogen detection, biomarker detection can also help to screen the categories of infectious pathogens. C-reactive protein and procalcitonin are used as bacterial biomarkers to identify bacterial infection and guide the use of antibiotics (Irwin et al., 2017; Katz, Sartori & Williams, 2019).

Is there viral biological indicator to identify viral infection and improve the accuracy of clinical diagnosis? *β*2-MG is a nonglycosylated protein (11.6 kDa) on the surface of almost all nucleated cells (Becker & Reeke Jr, 1985; Cunningham et al., 1973). *β*2-MG is a light chain of major histocompatibility complex class I, which plays a key role in adaptive immune system (Li, Dong & Wang, 2016; Wieczorek et al., 2017). Our results showed that the level of *β*2-MG in viral infection was significantly higher than that in bacterial infection. ROC curve analysis showed that *β*2-MG had high sensitivity and specificity in distinguishing viral from bacterial infection. This study was consistent with our previous study, and elevated *β*2-MG concentration might help to distinguish viral from bacterial infections in children with lower respiratory tract infection (Cai et al., 2020). The change of *β*2-MG level may be helpful in screening viral infection and avoiding antibiotic abuse. Another interesting result is that the level of *β*2-MG in children with exanthem subitum is significantly higher than that in children with viral URTI. We measured the concentration of *β*2-MG before the onset of rash in exanthem subitum. Therefore, this finding may help us to identify exanthem subitum early. We speculated that a significantly elevated serum *β*2-microglobulin level might indicate viral infection.

There are some limitations in this study. First, we only collected a small sample size of cases for study. Second, we only detected common pathogens, and did not exclude the combination of other undetectable pathogens, which would affect the results. Third, *β*2-MG detection has been routinely tested in clinic and is often used to assess renal function. The renal function of children is not mature until they were close to adults at 12 months old (Kearns et al., 2003). The challenge is that the effect of renal maturation on *β*2-MG needs further study.

## Conclusions

Serum *β*2-MG may be a significant biological indicator in children with acute URTI and exanthem subitum. Whether *β*2-MG can be used as a biomarker of viral infection is worthy of further exploration. Further studies are needed to confirm the reliability of the results in more patients and infectious diseases.

##  Supplemental Information

10.7717/peerj.11109/supp-1 Supplemental Information 1 *β*2-MG for discriminating adenovirus infection from bacterial infection

10.7717/peerj.11109/supp-2 Supplemental Information 2 Clinical characteristics and *β*2-MG levels of pathogens

10.7717/peerj.11109/supp-3 Supplemental Information 3 Original data

## Additional Information and Declarations

### Data Availability

The following information was supplied regarding data availability: Raw measurements are available in the Supplementary Files.
