# Supplementary figures and images for: Serum β2-microglobulin may be a viral biomarker by analyzing children with upper respiratory tract infections and exanthem subitum: a retrospective study
Source: PeerJ. 2021 Apr 6;9:e11109. doi: 10.7717/peerj.11109 (PMC8034339; doi:10.7717/peerj.11109)

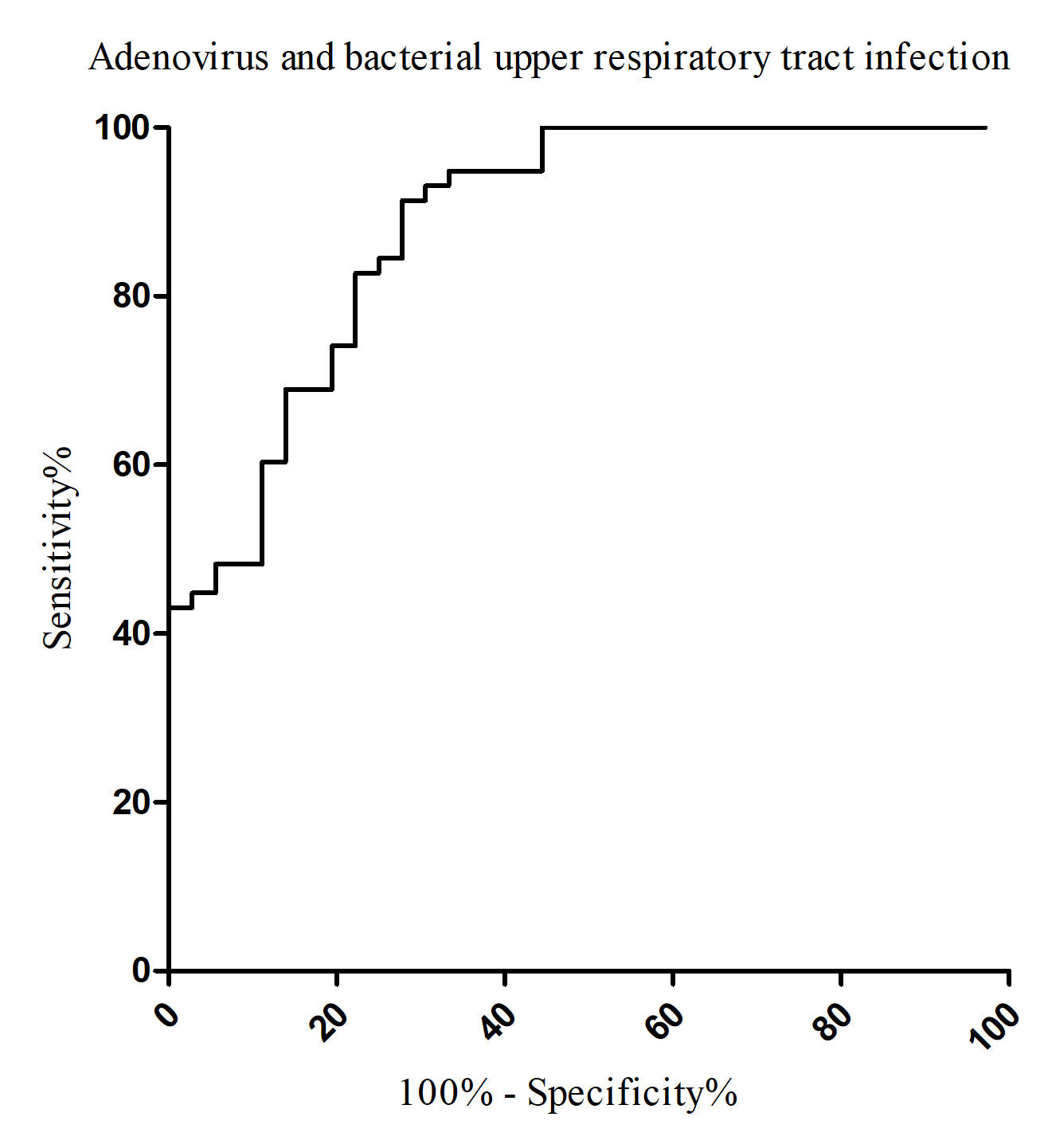

Supplement: Supplemental Information 1 [file peerj-09-11109-s001.jpg]
