# Supplementary material for: Serum β2-microglobulin may be a viral biomarker by analyzing children with upper respiratory tract infections and exanthem subitum: a retrospective study
Source: PeerJ. 2021 Apr 6;9:e11109. doi: 10.7717/peerj.11109 (PMC8034339; doi:10.7717/peerj.11109)
Supplement: Supplemental Information 2 [file peerj-09-11109-s002.docx]

| Characteristic | Streptococcus hemolyticus (n=22) | Staphylococcus haemolyticus (n=7) | Streptococcus pneumoniae (n=5) | Escherichia coli (n=2) | Influenza A virus (n=60) | Adenovirus (n=58) | Influenza B virus (n=37) | Parainfluenza virus (n=28) | Respiratory syncytial virus (n=14) | exanthem subitum (n=58) |
| --- | --- | --- | --- | --- | --- | --- | --- | --- | --- | --- |
| Swelling of tonsil, no. (%) | 19(86.4) | 1(14.3) | 2(40.0) | 0 | 17(28.3) | 34(58.6) | 13(35.1) | 5(17.9) | 3(21.4) | 6(10.3) |
| WBC,×10^9^/L |  |  |  |  |  |  |  |  |  |  |
| WBC＜4, no. (%) | 0 | 0 | 0 | 0 | 4(6.7) | 1(1.7) | 15(40.5) | 0 | 0 | 16(27.6) |
| 4≤WBC≤10, no. (%) | 7(31.8) | 1(14.3) | 0 | 0 | 41(68.3) | 20(34.5) | 17(45.9) | 17(60.7) | 8(57.1) | 36(62.1) |
| WBC＞10, no. (%) | 15(68.2) | 6(85.7) | 5(100) | 2(100) | 15(25.0) | 37(63.8) | 5(13.5) | 11(39.3) | 6(42.9) | 6(10.3) |
| CRP, mg/L |  |  |  |  |  |  |  |  |  |  |
| CRP≤10, no. (%) | 8(36.4) | 1(14.3) | 2(40.0) | 0 | 48(80.0) | 12(20.7) | 34(91.9) | 25(89.3) | 13(92.9) | 50(86.2) |
| CRP＞10, no. (%) | 14(63.6) | 6(85.7) | 3(60.0) | 2(100) | 12(20.0) | 46(79.3) | 3(8.1) | 3(10.7) | 1(7.1) | 8(13.8) |
| β2-MG Mean ± SD, mg/L | 2.00±0.52 | 1.89±0.40 | 1.56±0.36 | 2.07,1.95 | 3.04±0.84 | 2.72±0.48 | 2.91±0.45 | 3.02±0.74 | 2.94±0.55 | 4.19±0.90 |

Supplementary Table 1 Clinical characteristics and β2-MG levels of pathogens

Abbreviations: WBC, white blood cell; CRP, C-reactive protein; IQR, quartile range; β2-MG,β2-microglobulin

Percentages may not total 100 because of rounding.
